# Microbial carbon mineralization in tropical lowland and montane forest soils of Peru

**DOI:** 10.3389/fmicb.2014.00720

**Published:** 2014-12-18

**Authors:** Jeanette Whitaker, Nicholas Ostle, Niall P. McNamara, Andrew T. Nottingham, Andrew W. Stott, Richard D. Bardgett, Norma Salinas, Adan J. Q. Ccahuana, Patrick Meir

**Affiliations:** ^1^Centre for Ecology & Hydrology, Lancaster Environment CentreLancaster, UK; ^2^Soil and Ecosystem Laboratory, Lancaster Environment Centre, Lancaster UniversityLancaster, UK; ^3^School of Geosciences, University of EdinburghEdinburgh, UK; ^4^Faculty of Life Sciences, The University of ManchesterManchester, UK; ^5^Seccion Química, Pontificia Universidad Católica del PerúLima, Peru; ^6^School of Geography and the Environment, Environmental Change Institute, University of OxfordOxford, UK; ^7^Facultad de Ciencias Biologicas, Universidad Nacional de San Antonio AbadCusco, Peru

**Keywords:** soil organic matter, microbial community composition, decomposition, respiration, priming, cloud forest, ecosystem function

## Abstract

Climate change is affecting the amount and complexity of plant inputs to tropical forest soils. This is likely to influence the carbon (C) balance of these ecosystems by altering decomposition processes e.g., “positive priming effects” that accelerate soil organic matter mineralization. However, the mechanisms determining the magnitude of priming effects are poorly understood. We investigated potential mechanisms by adding ^13^C labeled substrates, as surrogates of plant inputs, to soils from an elevation gradient of tropical lowland and montane forests. We hypothesized that priming effects would increase with elevation due to increasing microbial nitrogen limitation, and that microbial community composition would strongly influence the magnitude of priming effects. Quantifying the sources of respired C (substrate or soil organic matter) in response to substrate addition revealed no consistent patterns in priming effects with elevation. Instead we found that substrate quality (complexity and nitrogen content) was the dominant factor controlling priming effects. For example a nitrogenous substrate induced a large increase in soil organic matter mineralization whilst a complex C substrate caused negligible change. Differences in the functional capacity of specific microbial groups, rather than microbial community composition *per se*, were responsible for these substrate-driven differences in priming effects. Our findings suggest that the microbial pathways by which plant inputs and soil organic matter are mineralized are determined primarily by the quality of plant inputs and the functional capacity of microbial taxa, rather than the abiotic properties of the soil. Changes in the complexity and stoichiometry of plant inputs to soil in response to climate change may therefore be important in regulating soil C dynamics in tropical forest soils.

## Introduction

Tropical forests are a globally significant store of terrestrial carbon (C) (Jobbagy and Jackson, 2000; Pan et al., 2011), but there is uncertainty as to the fate of this C in response to climate change (Wood et al., 2012; Cox et al., 2013). Climate change is expected to have complex indirect and direct effects on tropical forests, increasing plant productivity, modifying plant community composition and the resultant inputs of plant material to the soil, whilst also affecting decomposition and soil respiration rates (Chapin et al., 2009; Garcia-Palacios et al., 2012; Bardgett et al., 2013). The composite effect of these changes may alter the amount of organic C stored in tropical soils as this is determined by the balance between plant-derived inputs (litter and rhizodeposits) and outputs of C (root and soil respiration and dissolved organic carbon production). However, quantifying this C balance poses a critical challenge due to complex interactions between biological and physicochemical processes which mediate C inputs and losses in tropical soils (Billings and Ballantyne, 2013; Wieder et al., 2013).

There is growing experimental evidence and support from models that increased inputs of C to soil from leaf litter and rhizodeposits enhance soil respiration, and do not necessarily result in greater C storage (Sayer et al., 2011; Wieder et al., 2013; Xu et al., 2013). The mechanisms underlying this effect are not certain, but numerous studies show that labile C inputs from plants can accelerate the decomposition of older soil organic matter (SOM) pools, described as a “positive priming effect” (Kuzyakov, 2010). This has been demonstrated in soils of temperate (Bird et al., 2011; Sullivan and Hart, 2013) and arctic ecosystems (Hartley et al., 2012; Wild et al., 2014), and in lowland tropical and sub-tropical forests (Nottingham et al., 2012, 2015; Qiao et al., 2014). However, the biological mechanisms controlling the magnitude of priming effects are poorly resolved (Fontaine et al., 2011; Xu et al., 2014; Zhu et al., 2014), which hampers our ability to accurately predict the effects of climate change on soil C stocks (Cheng et al., 2014).

The magnitude and direction of priming effects appear to be influenced by both the composition of the soil microbial community and soil nutrient availability (Bird et al., 2011; Fontaine et al., 2011; Dijkstra et al., 2013; Pascault et al., 2013; Nottingham et al., 2015). Microbial species or taxa are known to differ in their functional properties and have distinct roles in the mineralization and assimilation of plant and SOM-derived C and nitrogen (N) resources (Schimel and Schaeffer, 2012; Waring et al., 2013), but it is unclear whether specific microbes are responsible for SOM mineralization as a result of priming effects (Kuzyakov, 2010; Chen et al., 2014). It has been shown that bacteria, specifically *r*-strategists, are the first to mineralize labile C entering the soil (Fontaine et al., 2003), accelerating the turnover of bacterial biomass and triggering apparent priming effects (Blagodatskaya and Kuzyakov, 2008). Beyond this, the role of specific microbial groups or taxa in any accelerated mineralization of SOM is poorly elucidated. In a small number of temperate grassland studies, gram-positive bacteria and fungi, which broadly correspond to a *K*-strategist classification (De Vries and Shade, 2013), were major controllers of rhizosphere priming (Fontaine et al., 2003, 2011; Bird et al., 2011; Dungait et al., 2013). In contrast, in lowland tropical forest soils no evidence was found that specific microbial groups were responsible for priming effects (Nottingham et al., 2012).

Two conceptual hypotheses have been proposed to describe the relationship between soil nutrient availability and SOM priming effects (Dijkstra et al., 2013). The “microbial mining” hypothesis posits that nutrient limitation increases SOM decomposition in response to labile C inputs. Thus, in soils of low nutrient availability, microbes are thought to use labile C as an energy source to extract nutrients from SOM, leading to positive priming effects (Moorhead and Sinsabaugh, 2006; Craine et al., 2007). On the other hand, the “preferential substrate utilization” hypothesis assumes that under high nutrient availability SOM mineralization decreases as microbes switch from SOM to labile C inputs for their C and energy requirements, leading to negative priming effects (Blagodatskaya et al., 2007).

The influence of microbial community composition and nutrient availability in regulating priming effects has not been tested in tropical montane forest soils, with only two studies in tropical lowland soils where priming effects were shown to be constrained by microbial N and phosphorus (P) limitation (Nottingham et al., 2012, 2015). Furthermore, research to date has not considered how the chemical complexity or stoichiometry of plant inputs interacts with soil nutrient availability to regulate priming effects. Here, we aimed to redress this lack of knowledge by identifying the microbial pathways by which C inputs and SOM are assimilated and respired, and the underlying mechanisms differentiating priming effects in lowland and montane tropical forest soils. For this, we used soils taken from a well-characterized tropical elevation gradient in the Peruvian Andes (Malhi et al., 2010), which transitions from lowland soils of low total C content and P availability and low abundance of fungi relative to bacteria, to montane soils of greater C content, with greater amounts of easily decomposable C, lower N availability, and a greater proportion of fungi relative to bacteria (Van De Weg et al., 2009; Zimmermann et al., 2012; Fisher et al., 2013; Turner and Wright, 2014; Whitaker et al., 2014). Moreover, using this gradient we previously showed that the relative abundance of microbial groups, specifically the ratio of fungi to bacteria and gram positive to gram-negative bacteria, was an important determinant of soil respiration responses to changing C inputs (Whitaker et al., 2014). Given these differences in N and P availability along this gradient, and the proposed significant role of fungi in priming effects (Fontaine et al., 2011), we anticipated that different priming mechanisms might operate along this gradient of lowland-to-montane forest soils. We hypothesized that: (H1) SOM mineralization as a result of priming will increase with elevation, consistent with microbial mining theory, due to increasing N limitation and the increased relative abundance of K-strategists; (H2) the magnitude and direction of priming effects are a function of substrate quality, with nitrogenous and labile C inputs inducing negative and positive priming effects, respectively; and (H3) microbial community composition will influence SOM mineralization through priming due to the functional capacity of *K*-strategists to mineralize both fresh plant inputs and SOM.

## Materials and methods

### Study site and field sampling

Soils were sampled in January 2012 from 10 sites along a tropical elevation gradient located on the east flank of the Peruvian Andes, with site elevations ranging from 210 to 3400 m asl (above sea-level) (Table 1). All the sites have continuous forest cover ranging from lowland Amazonian rainforest to upper montane cloud forest; the tree-line is situated at approximately 3500 m asl (Zimmermann et al., 2010b). Annual temperature decreases with increasing elevation along this transect, from 26 to 8°C whilst annual precipitation ranges from 1700 to 3200 mm yr^−1^ with maximum rainfall at mid-elevation (Table 1). Soil water content has been reported to have no clear relationship with elevation, partly due to limited evapotranspiration and fog deposition within the cloud immersion zone (1500–3400 m asl) (Van De Weg et al., 2009; Zimmermann et al., 2010a). Details of the vegetation, underlying geology, soil classification, aspect and slope are published elsewhere (Quesada et al., 2010; Zimmermann et al., 2010a; Rapp et al., 2012; Asner et al., 2014; Whitaker et al., 2014). At each site, the organic layer was sampled from 5 sub-plots located at random within established 1 ha plots. The organic layer was removed from a 40 × 40 cm area in each sub-plot to the maximum available depth, ranging from 2.5 to 22.8 cm (Table 2). Soils were sealed in plastic bags and transported to the laboratory in the UK where they were homogenized by mixing thoroughly by hand with large stones and woody debris removed, then stored at 4°C for a maximum of 4 weeks until used for experimentation and analysis.

**Table 1:** **Site characteristics along the tropical forest elevation gradient**.

**Site name**	**Site code**	**Elevation (m asl)**	**Latitude**	**Longitude**	**Mean annual temp (°C)**	**Annual precipitation (mm yr^−1^)**
Explorer's Inn 3	TAM-05	210	−12.830	−69.271	26.4	3199
Villa Carmen	VC	1000	−12.866	−71.401	20.7 ± 0.02	3087
San Pedro 2	SPD-02	1500	−13.049	−71.537	17.4 ± 1.5	2631
Trocha Union 8	TRU-08	1850	−13.071	−71.555	16.0 ± 1.3	2472
Trocha Union 7	TRU-07	2020	−13.074	−71.559	14.9 ± 1.1	1827
Trocha Union 5	TRU-05	2520	−13.094	−71.574	12.1 ± 1.0	na
Trocha Union 4	TRU-04	2720	−13.107	−71.589	11.1 ± 1.0	2318
Wayqecha	WAY-01	3025	−13.190	−71.587	11.1 ± 1.2	1706
Trocha Union 2	TRU-02	3200	−13.111	−71.604	8.9 ± 1.0	na
Trocha Union 1	TRU-01	3400	−13.114	−71.607	7.7 ± 1.1	2555

na, data not available.

**Table 2:** **Abiotic properties of 10 tropical forest soils from the elevation gradient**.

**Elevation (m asl)**	**Organic layer depth**	**Bulk density (g dwt cm^−3^)**	**Soil pH**	**Total soil C (% dwt)**	**Total soil N (% dwt)**	**Soil C:N**
210	2.5	0.52 (0.10)	4.11 (0.07)	8.6 (0.9)	0.7 (0.05)	13.1 (0.4)
1000	3.6	0.17 (0.03)	3.88 (0.07)	40.0 (1.7)	2.5 (0.04)	15.8 (0.6)
1500	16.0	0.18 (0.05)	4.62 (0.07)	49.0 (1.1)	2.8 (0.10)	17.6 (0.7)
1850	15.6	0.10 (0.02)	4.17 (0.06)	37.4 (4.2)	2.2 (0.23)	17.4 (1.1)
2020	16.8	0.16 (0.02)	4.12 (0.11)	44.2 (5.8)	2.2 (0.22)	19.8 (1.2)
2520	13.6	0.15 (0.01)	3.93 (0.16)	41.9 (5.2)	2.6 (0.26)	16.2 (0.7)
2720	21.4	0.18 (0.03)	3.85 (0.04)	49.5 (0.9)	2.7 (0.04)	18.3 (0.4)
3025	22.8	0.11 (0.01)	4.01 (0.12)	51.8 (0.3)	2.7 (0.10)	19.5 (0.7)
3200	11.8	0.23 (0.02)	4.03 (0.10)	49.7 (1.1)	3.2 (0.13)	15.6 (0.9)
3400	14.0	0.12 (0.02)	4.10 (0.11)	39.9 (6.3)	2.5 (0.31)	15.5 (1.5)
R-Sq		0.278	0.030	0.370	0.407	0.082
F		18.51	1.48	28.26	32.96	4.30
*P*		<0.0001	0.229	<0.0001	<0.0001	0.043

Samples were taken from the organic layer and data represent mean (SE) (n = 5). Relationships between soil properties and elevation analyzed by linear regression (R project). Non-normal data were square root transformed and checked for normality and homogeneity of variance prior to analysis.

### Analysis of soil abiotic properties

Fresh soil samples were analyzed for pH (soil: H_2_O, 1:2.5 w:v), gravimetric moisture content (dried at 105°C, to constant mass) and bulk density (Emmett et al., 2008). Dried, ground soil samples (approximately 100 mg) were analyzed for total C and N content using a TruSpec CN Elemental Determinator (LECO, St Joseph, Michigan, USA). Maximum water holding capacity (WHC) was calculated on composite soil samples for each elevation (composite of 5 sub-plots per site) as the amount of water remaining in the soil after being saturated and left to drain for 12 h in a fully humid airspace (Ohlinger, 1995).

### Microbial assimilation and mineralization of C inputs

Ten soils from the elevation gradient (Table 1) were incubated with four ^13^C labeled substrates for 7 days. Substrates of varied quality and ecological relevance were selected which had been used to investigate the importance of microbial community composition on C substrate mineralization (Whitaker et al., 2014). The compounds were: xylose (simple), vanillin (intermediate), and hemicellulose (complex) and glycine, a small amino acid containing C and N. Sub-plot soil samples (5) for each elevation were treated as individual replicates. All soils were adjusted to 75% of maximum WHC (allowing for the addition of the substrate in solution) and incubated with one of the substrates or a control treatment (sterile deionized water). Aliquots (5 g fwt) of each soil were placed in 175 ml Wheaton bottles. ^13^C enriched substrates (99 atom %) were mixed with equivalent ^12^C substrates to produce 10 atom% solutions. The substrates xylose, glycine, and vanillin (Sigma-Aldrich, Gillingham, UK) were diluted in sterile deionized water so that each substrate was added in 555 μl deionized water per incubation. The non-soluble substrate hemicellulose (IsoLife bv, Wageningen, The Netherlands) was diluted into suspension, sonicated for 10 min and vortexed for 5 s prior to pipetting and mixing into the soil. The final concentration of substrate added was 0.2 mg C g^−1^ soil fwt, equating to between 53 and 100% of initial microbial biomass C (Whitaker et al., 2014). This concentration was chosen in order to add sufficient C to increase soil respiration and induce changes in microbial activity and turnover, without inducing a significant increase in microbial growth (Blagodatskaya and Kuzyakov, 2008). Following substrate addition the headspace of each bottle was flushed with compressed air for 1 min (to achieve a standard starting atmosphere), bottles were then sealed with butyl rubber stoppers and aluminum crimp caps. Bottles were over-pressurized by injecting 20 ml of compressed air, to allow for subsequent headspace gas sampling, and were incubated for 7 days at 20°C in the dark. The headspace of each bottle was sampled at 24, 48, and 168 h by taking two 5 ml samples with an air-tight syringe and injecting them into two 3.5 ml exetainer vials (Labco, Lampeter, UK), for δ^13^C and concentration analyses of CO_2_. At the end of the experiment, soils were frozen at –80°C and freeze-dried for analysis of phospholipid fatty acid biomarkers (PLFAs) and for δ^13^C values of PLFAs.

### CO_2_ and ^13^C-CO_2_ analyses

CO_2_ samples were analyzed on a PerkinElmer Autosystem Gas Chromatograph (GC) fitted with a flame ionization detector containing a methaniser (Case et al., 2012). Results were calibrated against certified gas standards (BOC Ltd. Guildford, UK) and converted to a total CO_2_ flux reported as CO_2_-C (μg g soil dwt^−1^ d^−1^) (Holland et al., 1999). As fluxes were not linear over time for treated soils, exponential curves were fitted to the respiration data for each replicate bottle and fluxes calculated using these curves, calculating mean ± SE for the 5 replicates.

δ^13^C values of CO_2_ were measured using a trace-gas pre-concentrator coupled to an isotope ratio mass spectrometer (IRMS, Isoprime Ltd). Between 100 and 400 μl of headspace was introduced into the injection port of the pre-concentrator, whereupon water was removed by a magnesium perchlorate chemical trap and the CO_2_ cryogenically focused before entering the IRMS via an open split. The isotope ratio of the resultant CO_2_ was compared to pulses of known reference CO_2_. The instrument was calibrated on each day of analysis. Blanks were run prior to each analytical batch, in addition to a quality control reference CO_2_ standard and duplicate analysis after every 15th sample. Precision was greater than or equal to ± 0.2‰.

### PLFA and ^13^C-PLFA analysis

Soil microbial community composition was determined using phospholipid fatty acid (PLFA) analysis after the 7 day incubation period (Bardgett et al., 1996; De Deyn et al., 2011). Phospholipids were extracted from 1.5 g soil fresh weight for all 10 soils from the control treatments (5 replicates) and four selected soils (210, 1000, 2020, 3025 m asl) from the substrate treatments (3 replicates randomly selected). The number of substrate-amended soils and replicates was rationalized due to the cost and time required for compound specific PLFA analysis and soils were selected as representative of the range of site elevations and soil C contents of these soils. Identification of individual PLFAs was carried out using gas chromatography mass spectrometry (GC-MS) using an Agilent Technologies 5973 Mass Selective Detector coupled to an Agilent Technologies 6890 GC. Concentrations were calculated for all identifiable PLFAs via an internal standard (C19 FAME, Sigma-Aldrich). Gram-positive (GP) bacteria were identified by the terminal and mid-chain branched fatty acids (15:0i, 15:0a, 16:0i, 17:0i, 17:0a) and cyclopropyl saturated and monounsaturated fatty acids (16:1ω7, 7,cy-17:0, 18:1ω7, 7,8cy-19:0) were considered indicative of gram-negative (GN) bacteria (Rinnan and Baath, 2009). The fatty acids 18:2ω6,9 and 18:1ω9 were considered to represent saprotrophic and ectomycorrhizal (SP/ECM) fungi (Kaiser et al., 2010). Total PLFA concentration (μg g^−1^ soil dwt) was calculated from all identified PLFAs (15:0, 14:0, 16:0, 16:1, 16:1ω5, 16:0, 17:1ω8, 7Me-17:0, br17:0, br18:0, 18:1ω5, 18:0, 19:1; plus those listed above). The ratio of fungal to bacterial (F:B) PLFAs and GP to gram-negative bacteria (GP:GN) PLFAs were taken to represent the relative abundance of these microbial functional groups.

δ^13^C values of individual PLFA's from four of the treated soils (210, 1000, 2020, 3025 m asl) were analyzed using gas chromatography-combustion-isotope ratio mass spectrometry (GC-C-IRMS). Compounds were separated using an Agilent Technologies 6890 GC (splitless mode) with helium as the carrier gas. The GC effluent was diverted via a heart split union to a ceramic combustion furnace packed with a copper oxide/platinum/nichrome catalyst wire heated at 940°C. Water was subsequently removed from the combustion products by a passing the effluent through a nafion membrane, prior to the CO_2_ entering the IRMS (Isoprime Ltd, Manchester, UK). The PLFA δ^13^C values were corrected for the addition of the extra C atom introduced to the molecule during methylation, using a correction factor obtained by CF-EA-IRMS measurement on the derivatizing methanol and application of a mass balance equation (Jones et al., 1991).

### Stable isotope calculations

Enrichment of ^13^C in CO_2_ and individual PLFAs was expressed as δ^13^C (‰) which represents the ratios (R) of ^13^C:^12^C relative to the PDB standard (0.0112372) (Coleman and Fry, 1991) defined as:

$$\delta^{13}\text{C}=[({\text{R}}_{\text{sample}}-{\text{R}}_{\text{standard}})/{\text{R}}_{\text{standard}}]\times 1000$$

#### ^13^CO_2_ calculations

The percentage of respired CO_2_ derived from ^13^C substrate was calculated for control and treated soils at 0, 24, 48, and 168 h, with a mixing model according to:

$$\%{\text{C}}_{\text{substrate derived}}=[(\delta_{\text{C}}-\delta_{\text{T}})/(\delta_{\text{C}}-\delta_{\text{L}})]\times 100$$

Where δ_C_ is the δ^13^C value of the respired CO_2_ from control soils, δ_T_ is the δ^13^C value in respired CO_2_ from treated soils and δ_L_ is the δ^13^C value of the labeled substrate. These data were then used to calculate the substrate-derived C respired after 24, 48, and 168 h for all soil/substrate combinations, expressed in μg C g^−1^ soil dwt. The change in SOM-C utilization following substrate addition (positive or negative priming) was calculated as the total respiration (ppm) in treated soils minus control respiration (ppm) minus the substrate-derived respiration (ppm) calculated above and expressed as “Primed C” in μg C g^−1^ soil dwt.

#### ^13^C-PLFA calculations

Isotopic enrichment of individual PLFAs were expressed as δ^13^C values corrected for the methyl group added during methanolysis where:

$$\delta^{13}{\text{C}}_{\text{PLFA}}=[({\text{N}}_{\text{PLFA}}+1)\delta^{13}{\text{C}}_{\text{FAME}}-\delta^{13}{\text{C}}_{\text{MeOH}}]/{\text{N}}_{\text{PLFA}}$$

N_PLFA_ refers to the number of C-atoms of the PLFA molecule, δ^13^C_FAME_ is the measured δ^13^C value of the FAME after methylation and δ^13^C_MeOH_ is the δ^13^C value for the methanol used for methanolysis (−50.23‰). To calculate the percent of substrate-derived C within individual PLFAs we used a variation of equation 2 multiplied by PLFA abundance (mol %). The % substrate-derived C was calculated where δ_C_ is the δ^13^C value of a specific PLFA from control soils, δ_T_ is the δ^13^C value for the same PLFA from treated soils and δ_L_ is the δ^13^C value of the labeled substrate (Williams et al., 2006; Nottingham et al., 2009). These data were used to calculate the actual incorporation (μg PLFA-^13^C g^−1^ soil dwt) into individual and total PLFAs, and the proportional incorporation of substrates into individual PLFAs as a percentage of the total incorporated. These were then summed to compare the actual and proportional incorporation into key microbial groups [fungi (SP + ECM), bacteria, GP, and GN bacteria]. Microbial carbon-use efficiency (CUE) was calculated as the ratio of ^13^C assimilated into total PLFAs relative to that respired.

### Statistical analysis

All statistics were conducted using the statistical package R, version 2.14.0. To investigate whether soil properties or substrate quality determine the magnitude of priming (H1 and H2) we analyzed main and interactive effects of substrate, soil and time on differences in cumulative substrate-derived C, primed C, and their ratio by three-way analysis of variance. Data were square-root transformed (constant added to primed C data), and pair-wise comparisons of interactive effects were performed using Tukeys HSD *post-hoc* tests following One-Way ANOVA on soil, substrate, and time data subsets (Supplementary Tables 1, 2). To test how microbial community composition influenced the assimilation and respiration of C inputs (H3), we analyzed main and interactive effects of substrate and soil on microbial incorporation of ^13^C substrates using Two-Way ANOVA. Data analyzed included actual incorporation (μg ^13^C-PLFA g^−1^ soil dwt), the relative proportion (%) of ^13^C incorporated into microbial groups (fungi, GP and GN bacteria) and microbial CUE. Pair-wise comparisons of interactive effects were analyzed as described above.

## Results

### Soil abiotic and biotic properties

The tropical forest soils sampled between 210 m and 3400 m asl varied significantly in abiotic and biotic properties. Total C and N and soil C:N increased significantly with elevation, soil pH was unchanged, whilst bulk density decreased with elevation (Table 2). The lowland soil organic layer (210 m) was characterized by lower total C (9%) and N (0.7%) content and soil C:N ratio (13.1) and higher bulk density (0.5 g dwt cm^−3^) than the remaining sites, where values ranged from 37 to 52% C content, 2.2–3.2% N content, 15.5–19.8 C:N ratio and 0.10–0.23 g dwt cm^−3^ bulk density (Table 2).

Microbial community abundance and composition also changed across the gradient (Table 3). The abundance of total PLFAs (μg g^−1^ soil dwt) and all microbial PLFA biomarkers (total fungi and bacteria, GP and GN bacteria) increased significantly with elevation, as did the ratio of F:B, whilst the GP:GN ratio decreased with elevation. The lowland soil (210 m) had the lowest concentration of total PLFAs and all microbial groups, the lowest F:B ratio and the highest GP:GN ratio (Table 3).

**Table 3:** **Microbial community abundance and composition of 10 tropical forest soils**.

**Elevation (m asl)**	**Total PLFA**	**Fungal PLFA**	**Bacterial PLFA**	**GP PLFA**	**GN PLFA**	**F:B PLFA**	**GP:GN PLFA**
	**μg PLFA g^−1^ soil dwt**						
210	167.7 (2.4)	11.6 (1.1)	92.8 (6.4)	55.1 (2.2)	35.7 (7.7)	0.13 (0.01)	1.56 (0.03)
1000	597.9 (36.3)	58.4 (8.1)	311.9 (46.2)	171.2 (7.7)	135.4 (39.6)	0.19 (0.03)	1.29 (0.22)
1500	510.1 (68.4)	78.8 (19.7)	224.8 (38.0)	95.7 (8.9)	125.5 (30.7)	0.34 (0.02)	0.77 (0.18)
1850	511.2 (22.3)	57.3 (6.8)	254.3 (33.9)	113.3 (13.7)	137.1 (20.1)	0.23 (0.01)	0.83 (0.09)
2020	533.3 (110.3)	50.3 (9.7)	314.3 (16.3)	173.5 (17.1)	137.8 (8.9)	0.16 (0.03)	1.23 (0.13)
2520	794.8 (162.5)	86.6 (24.4)	414.3 (22.5)	168.3 (20.3)	241.3 (5.0)	0.20 (0.03)	0.73 (0.12)
2720	767.9 (5.3)	95.9 (3.9)	382.9 (15.9)	165.1 (9.1)	213.1 (7.3)	0.25 (0.03)	0.78 (0.03)
3025	733.1 (75.5)	100.0 (10.2)	313.0 (16.9)	168.5 (8.5)	138.3 (8.6)	0.32 (0.02)	1.24 (0.02)
3200	640.0 (51.4)	78.9 (12.3)	315.7 (17.8)	139.3 (6.2)	172.6 (11.8)	0.25 (0.03)	0.81 (0.03)
3400	641.1 (2.8)	87.2 (15.1)	292.6 (6.6)	138.6 (0.6)	149.6 (6.6)	0.30 (0.06)	0.93 (0.04)
R-Sq	0.465	0.467	0.338	0.222	0.351	0.238	0.264
F	23.43	23.69	13.77	7.69	14.63	8.43	9.71
*P*	<0.0001	<0.0001	0.0009	0.010	0.0007	0.007	0.004

Samples were taken from the organic layer and data represent mean (SE) (n = 5). Relationships between soil properties and elevation analyzed by linear regression (R project). Non-normal data were square root transformed and checked for normality and homogeneity of variance prior to analysis. F, fungal; B, bacterial; GP, gram-positive bacteria, and GN, gram-negative bacteria.

### Microbial mineralization of ^13^C substrates

**Figure 1:**
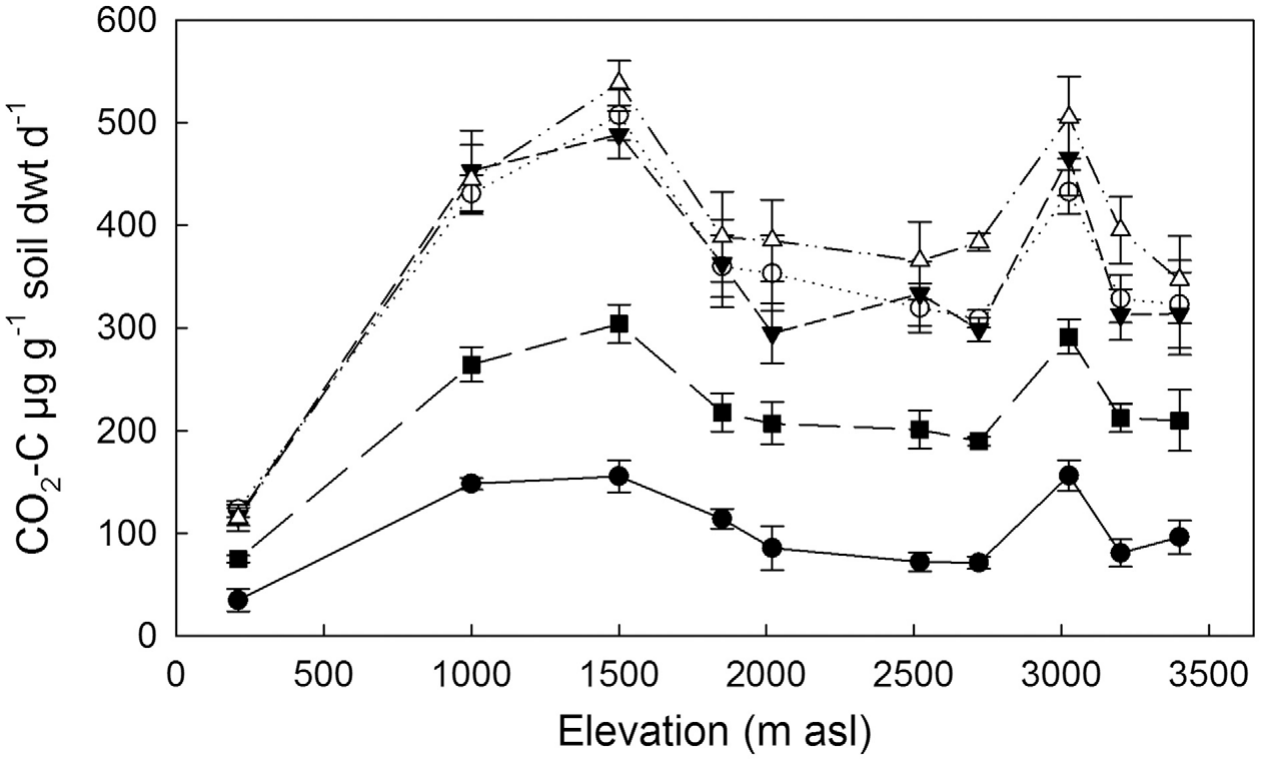
**Basal and substrate-induced respiration of 10 tropical forest soils incubated at standard temperature (20°C) and moisture (75% max. WHC).** Data represent mean ± SE (*n* = 5). Control (•), xylose (◦), vanillin (∆), hemicellulose (■), glycine (▼).

Basal respiration of the 10 organic soils, incubated under controlled conditions, varied approximately four-fold (35–157 μg CO_2_-C g^−1^ soil dwt d^−1^) with lowest fluxes (on a soil mass basis) from the 210 m soil and highest fluxes from the 1000, 1500, and 3025 m soils (Figure 1). Increases in respiration in response to C inputs varied among substrates in all soils, with the smallest fluxes in response to hemicellulose (Figure 1). Responses to the other three substrates were greater and not significantly different in magnitude from each other for each soil. The lowest substrate-induced fluxes came from the lowland soil, but there was no trend with elevation among the remaining soils.

**Figure 2:**
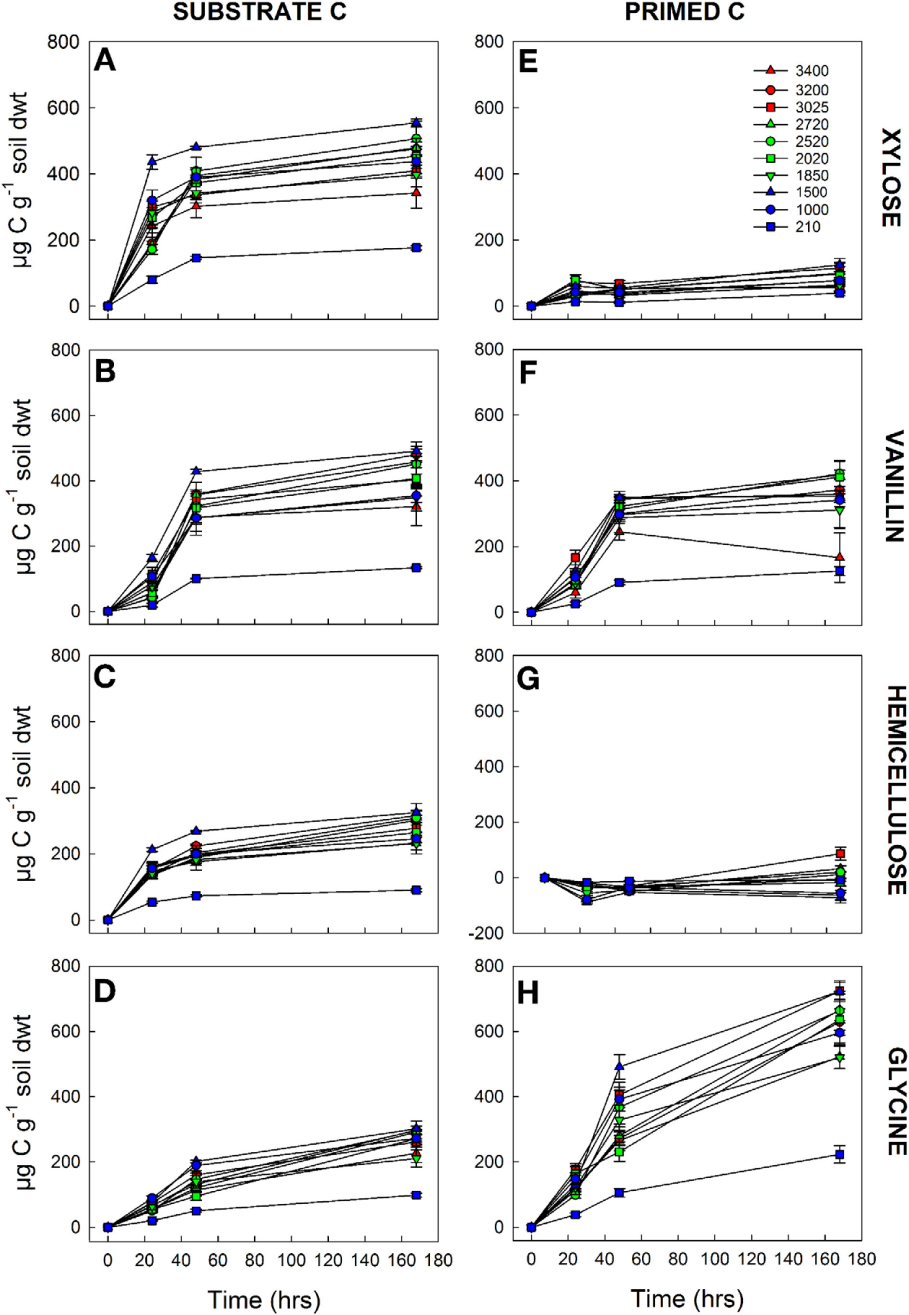
**Mineralization of four C substrates [xylose (A,E), vanillin, (B,F), hemicellulose (C,G), glycine (D,H)] by 10 tropical forest soils (elevation m asl).** Comparison of cumulative substrate C **(A–D)** and primed C **(E–H)** (μg g^−1^ soil dwt) respired over 7 days at standard temperature (20°C) and moisture (75% max WHC). Data represent mean ± SE (*n* = 5). Three-Way anova is presented in Table 4 and Supplementary Tables 1, 2.

Using an isotopic source partitioning approach we determined the origin of any additional respiration (above basal) at each time point, i.e., CO_2_ derived either from the ^13^C substrate (substrate C) or from native SOM, hereafter termed “primed C” (Figure 2). The quantity of substrate C respired varied significantly among the 10 soils and with substrate quality, with significant interactions between soil × substrate, soil × time, and substrate × time (Table 4). After 168 h incubation, a significantly smaller amount of hemicellulose and glycine had been mineralized in all soils than vanillin or xylose (Figures 2A–D). The amount of substrate respired decreased with increasing substrate recalcitrance (xylose > vanillin > hemicellulose) for the C only compounds, and the mineralization of glycine (C + N) was significantly lower (Figures 2A–D). This relationship with substrate quality was consistent among all 10 soils, despite significant differences between soils in the overall amount of substrate respired (Figures 2A–D and Table 4). *Post-hoc* tests of the soil × substrate interaction revealed that after 168 h incubation, the lowest and highest elevation soils were distinct from the remaining soils: for example these two soils mineralized the smallest amount of xylose and vanillin (Figures 2A,B and Supplementary Table 1).

**Table 4:** **Differences in respired substrate-derived C, primed C and their ratio after incubation with four substrates of varying quality in organic soils from an elevation gradient**.

	**Term**	***F***	***P***
Substrate C	Soil	102.78	<0.0001
	Substrate	385.66	<0.0001
	Time	858.31	<0.0001
	Soil × substrate	2.06	0.0016
	Soil × time	6.14	<0.0001
	Substrate × time	60.96	<0.0001
	Soil × substrate × time	0.94	0.604
Primed C	Soil	26.97	<0.0001
	Substrate	1469.01	<0.0001
	Time	462.53	<0.0001
	Soil × substrate	10.57	<0.0001
	Soil × time	4.00	<0.0001
	Substrate × time	114.23	<0.0001
	Soil × substrate × time	2.14	<0.0001
Primed: substrate C	Soil	14.27	<0.0001
	Substrate	4231.14	<0.0001
	Time	2.83	0.06
	Soil × substrate	5.68	<0.0001
	Soil × time	3.91	<0.0001
	Substrate × time	48.37	<0.0001
	Soil × substrate × time	2.29	<0.0001

Data were square-root transformed (constant added to primed C data) and analyzed by Three-Way anova with soil, substrate, and time as factors. Pair-wise comparisons were performed by Tukeys HSD following One-Way anova on data subset by site, substrate, and time (Supplementary Tables 1, 2).

The amount of primed C varied significantly among soils and substrates with a significant soil × substrate × time interaction (Table 4 and Figures 2E–H). Substrate identity had the greatest effect on primed C: hemicellulose induced a negative priming effect i.e., a reduction in SOM-mineralization compared to the control, which persisted longer in lower elevation soils; xylose induced a small positive priming effect; vanillin induced an equivalent amount of primed C to that which was substrate-derived; whilst glycine induced the largest overall release of primed C (Figures 2E–H). Priming was lower in the lowland (210 m) soil than the remaining soils for xylose, vanillin, and glycine over the duration of the incubation, although differences were not significant for all soils/time points with the exception of glycine (Figures 2E,F,H and Supplementary Table 2).

**Figure 3:**
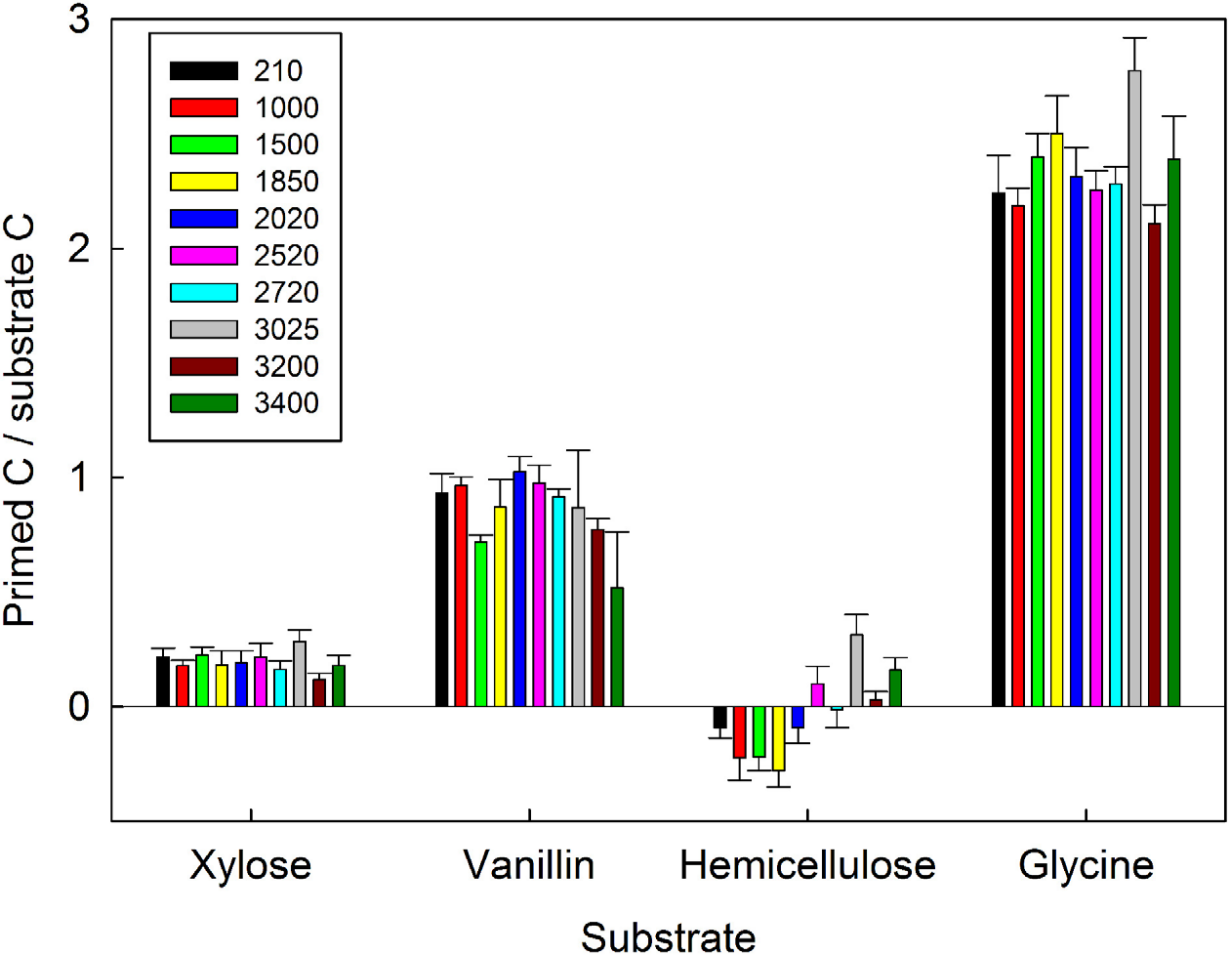
**The proportion of primed C relative to substrate C respired by 10 tropical forest soils (elevation m asl) after 168 h incubation with four C substrates (xylose, vanillin, hemicellulose, glycine).** Data represent mean ± SE (*n* = 5). Three-Way anova is presented in Table 4.

The ratio of primed to substrate C was extremely consistent for each substrate among all soils, regardless of differences in soil properties (Figure 3). These data demonstrate that whilst the increase in microbial respiration in response to glycine, vanillin, and xylose additions was similar in magnitude for each soil (Figure 1), the source of the respired C was significantly different for the four substrates and changed over time (soil × substrate × time interaction, *P* < 0.0001, Figure 3). Over the 7 day incubation, primed C was the source of 16% of the additional flux in response to xylose (Figures 2A,E) and 46% for vanillin (Figures 2B,F), whilst in the glycine amended soil, primed C was the source of 70% of the total additional flux, with primed C 2.4-fold greater than substrate C (Figures 2D,H, 3). Increased respiration in response to hemicellulose was predominantly all substrate-derived with a small suppression of SOM mineralization, i.e., negative priming, in the majority of soils at 24 and 48 h, whilst after 168 h there was a small positive priming effect in soils from above 2020 m elevation (Figures 2, 3).

### Fate of C substrates in microbial communities

**Figure 4:**
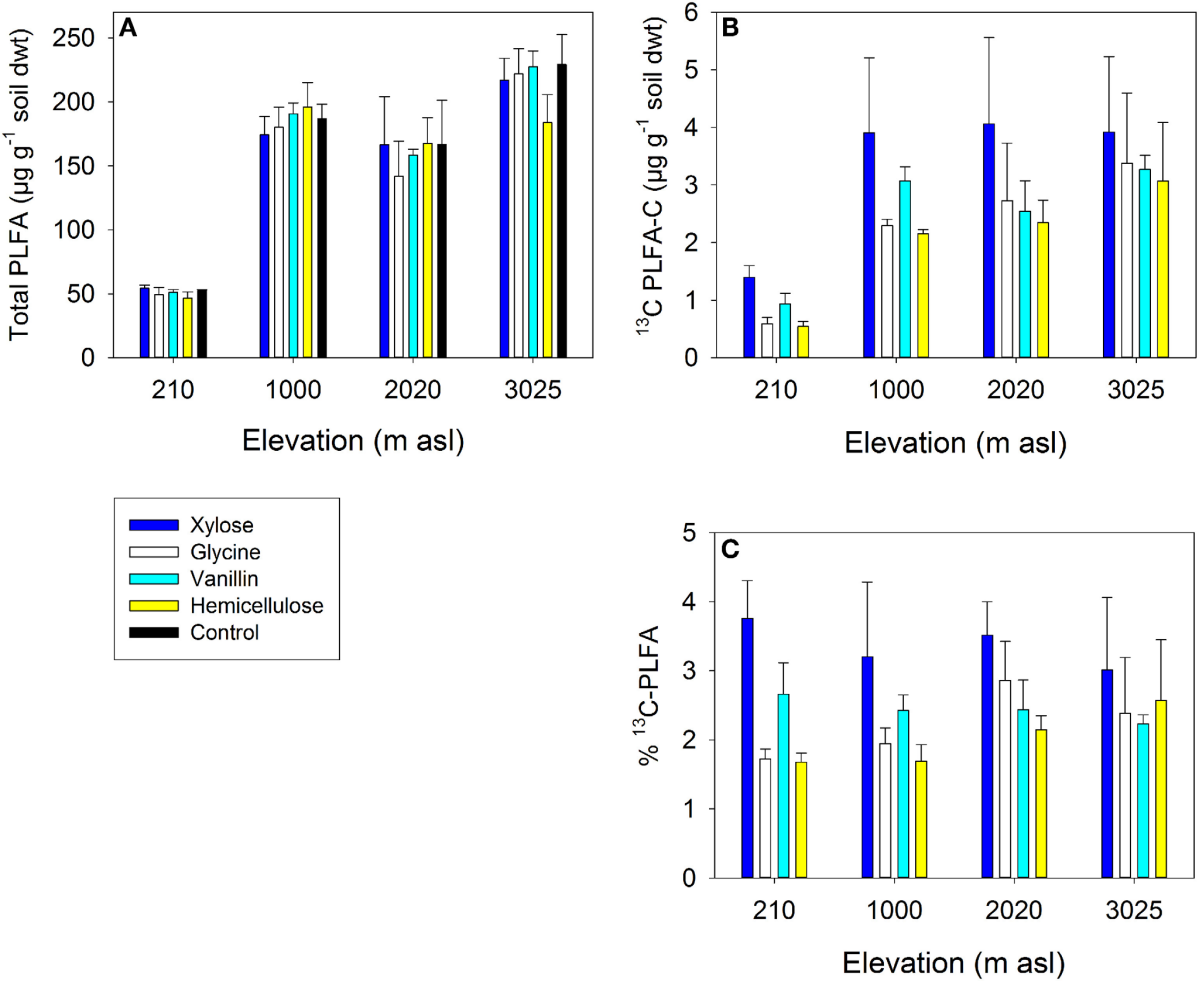
**Microbial abundance and assimilation of ^13^C substrates into PLFAs in four tropical forest soils after 7 days incubation: (A) Total PLFA concentration (B) ^13^C incorporation into PLFAs (μg g^−1^ soil dwt) (C) % of PLFA-C derived from substrate.** Data represent mean ± SE (*n* = 3). Two-Way anova is presented in Table 5.

The fate of ^13^C substrates in PLFA biomarkers (total PLFAs and key microbial groups) varied significantly between soils and substrates (Table 5). Soil (i.e., site elevation) explained a greater proportion of the variance in total ^13^C incorporation by PLFAs than did substrate identity (Table 5). There was significantly less ^13^C assimilated into PLFAs in the lowland soil (210 m) compared to the other soils for all four substrates, which was a function of the smaller size of the microbial community in this soil (Table 5 and Figure 4). There was also typically lower total incorporation of glycine and hemicellulose compared with vanillin and xylose for any individual soil; this was consistent with the lower mineralization of these substrates indicated from respiration (Figures 3, 4C). As expected, substrate addition did not significantly increase the microbial biomass of the soil, as indicated by total PLFA (Figure 4A, see Section Microbial Assimilation and Mineralization of C Inputs).

**Table 5:** **Effects of soil and substrate identity on the fate of substrate-C in microbial PLFA biomarkers: actual incorporation (μg ^13^C-PLFA g^−1^ soil dwt), % of PLFA-C which is substrate-derived, proportion (%) of ^13^C assimilated into specific microbial groups; and microbial CUE (total ^13^C-PLFA incorporation relative to substrate respired ^13^C**.

**Incorporation measure**	**PLFA measure**	**Soil**	**Substrate**	**Soil × Substrate**
Actual	Total ^13^C-PLFA (μg g^−1^ soil dwt)	^***^ (46.87)	^**^ (6.83)	ns
	% PLFA-C substrate-derived	ns	^***^ (12.19)	ns
Proportion (%)	GP	^**^ (5.27)	^***^ (161.29)	ns
	GN	^***^ (8.40)	^***^ (58.13)	^*^ (2.71)
	Fungal	^***^ (13.48)	^*^ (3.71)	^**^ (3.70)
Microbial CUE	total PLFA^13^C/respired ^13^C	^**^ (5.56)	ns	ns

Data were analyzed by Two-Way anova with post-hoc pair-wise comparisons with Tukeys HSD, values represent P-value (F statistic) where

**Figure 5:**
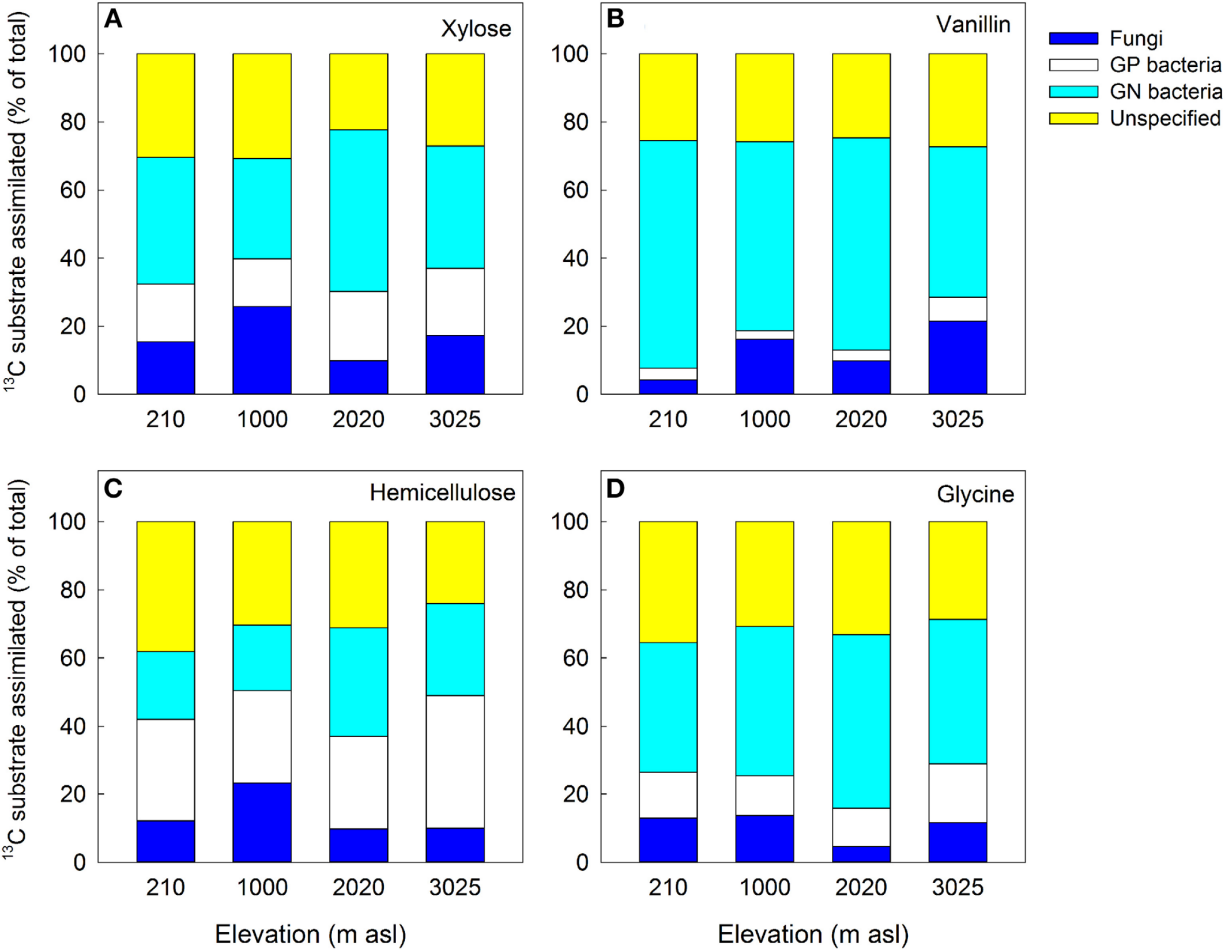
**Proportion of ^13^C substrate assimilated into biomarker PLFAs of key microbial groups (% of total incorporation into all PLFAs): (A) xylose; (B) vanillin; (C) hemicellulose and (D) glycine.** Fungi = SP + ECM fungi; GN = gram-negative bacteria; GP = gram-positive bacteria; Unspecified = unspecified microbial PLFAs. Data represent mean ± SE (*n* = 3). Two-Way anova is presented in Table 5.

**Figure 6:**
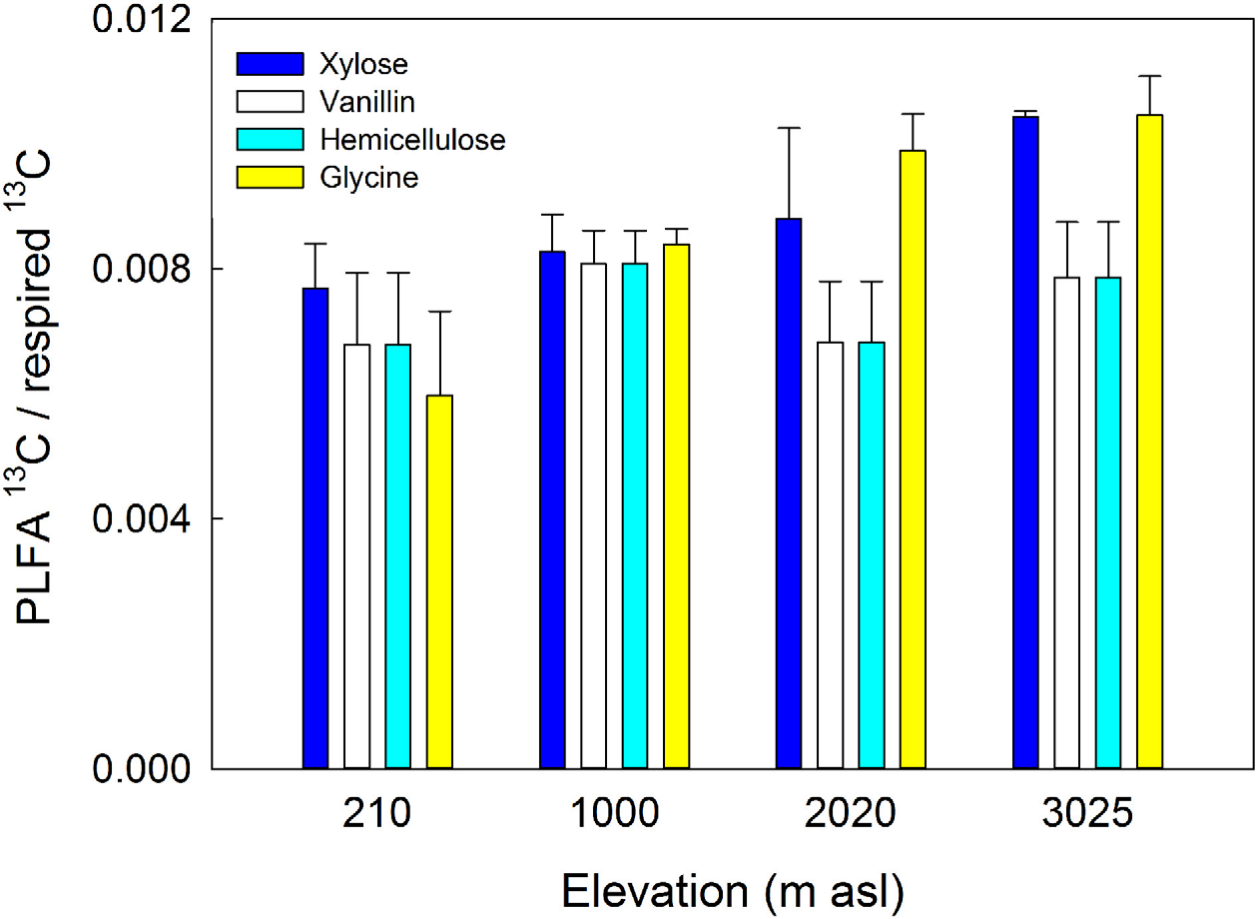
**Microbial carbon use efficiency (^13^C substrate incorporation into PLFAs relative to respired ^13^C-substrate) in four gradient soils in response to four C substrates.** Data represent mean ± SE (*n* = 3). Two-Way anova is presented in Table 5.

* , P < 0.05;

** , P < 0.01;

*** , P < 0.01; ns, not significant. Data illustrated in Figures 4–6.

The distribution of the ^13^C substrates between microbial groups also differed significantly between soils and substrates, with significant main effects of soil and substrate for GP bacteria and a significant soil × substrate interaction for GN bacteria and fungi (Table 5). For GP and GN bacteria, substrate was most important in explaining the variance in substrate incorporation; for example GP bacteria assimilated a very low proportion of vanillin and a high proportion of hemicellulose in all four soils compared to other microbial groups, whilst GN bacteria showed the reverse pattern (Figure 5). In contrast, the greatest differences in fungal assimilation were among soils: lowest incorporation of vanillin was in the 210 m soil and lowest incorporation of glycine in the 2020 m soil (Figure 5 and Table 5).

The amount of substrate ^13^C incorporated into total PLFAs relative to that respired differed significantly between soils (*P* = 0.044) but not substrates, with a significantly lower ratio in soils from 210 m to 1000 m asl compared to soil from 3025 m asl (Table 5 and Figure 6). As an indicator of microbial CUE (microbial assimilation/respiration), this suggests that microbial CUE was greater at high compared to low elevation, but substrate quality did not affect microbial CUE.

## Discussion

Changes in plant productivity and plant community composition are expected across the globe as a result of climate change, with the potential for alterations in the soil C balance. In order to predict any climate-C cycle feedbacks which might result we need to better understand the complex biogeochemical processes, mediated by the soil microbial community, which regulate the mineralization of fresh plant inputs and older SOM pools. Using C substrates as surrogates for plant inputs, our results show that substrate quality, defined by complexity and stoichiometry, was the single strongest factor controlling the balance between substrate mineralization and priming of SOM decomposition (Figure 3). Our measurements suggest that this was due to functional differences in key soil microbial groups that influenced mineralization of labile and recalcitrant C sources. Changes in the complexity and stoichiometry of plant inputs to soil in response to climate change may therefore be important in regulating soil C dynamics in tropical forest soils.

### Effects of soil properties and substrate complexity on priming effects

We expected that the magnitude of priming effects would increase with elevation due to decreasing N availability and an increased abundance of *K*-strategists (H1). We found that whilst the amount of substrate C and primed SOM-C respiration varied significantly between soils (Table 4), there were no consistent trends with elevation or soil properties. Furthermore, the ratio of primed C: substrate C was consistent in soils from all elevations (Figure 3). This partially disproves our first hypothesis (that the magnitude of priming effects would increase with elevation) and contradicts research in temperate agricultural soils where priming effects were shown to be soil-specific and dependent on soil C content (Paterson and Sim, 2013). These results suggest a negligible effect of soil nutrient availability on priming of SOM mineralization in contrast to a study in lowland tropical forest soils, where priming was promoted in intermediate fertility soils, but constrained in low and high fertility soils (Nottingham et al., 2012). This possibly reflects the combined effects of decreasing N availability in tandem with increasing P availability in soils along the elevation gradient (Cleveland et al., 2006; Bradford et al., 2008a; Cusack et al., 2011; Dijkstra et al., 2013; Turner and Wright, 2014). However, further information on the different forms of P in soils along this gradient is needed in order to more fully understand the interactions between these limiting macronutrients and their role in regulating priming effects.

Using an isotopic source partitioning approach we observed that similar increases in respiration in response to xylose, vanillin and glycine masked major differences in the sources of respired C (Figure 1). Increases in respired C were typically derived from both substrate inputs and SOM (primed C) but with different relative magnitudes; negligible priming effects were observed in response to a complex C substrate (hemicellulose) whilst large priming effects were observed in response to an amino acid containing labile C and N (Figure 2 and Table 4). These findings, which were consistent among soils from all elevations, support our hypothesis (H2) that labile C inputs will induce greater positive priming effects compared to more complex C inputs with different mechanisms operating dependent on substrate complexity. The response to labile C inputs (xylose and vanillin) is consistent with microbial mining; microbes used labile C inputs (xylose and vanillin) as an energy source, in order to exploit nutrients from SOM (positive priming), but addition of the more recalcitrant substrate hemicellulose did not result in significant priming effects. Microbial mining has been demonstrated in laboratory experiments with agricultural soil where addition of sucrose was shown to accelerate SOM mineralization (Chen et al., 2014), and is supported by observations of increased activity of N-degrading enzymes associated with priming effects (Asmar et al., 1994). However, if microbial mining was driving the priming response in our study, we would have expected increased SOM decomposition with elevation corresponding with increasing N limitation. This wasn't the case and is most likely attributable to local differences in microbial N and P co-limitation along the gradient (Fisher et al., 2013; Nottingham et al., 2015).

We also hypothesized (H2) that addition of the amino acid, glycine, would cause negligible priming of SOM mineralization as a result of preferential utilization of this nitrogenous substrate. Unexpectedly and contrary to H2, we observed that glycine inputs resulted in a large positive priming effect and low glycine mineralization in soils from all elevations (Figures 2D,H). There are two potential explanations for this result. First, inputs of combined C and N may have stimulated microbial growth and/or turnover and exo-enzyme production, promoting increased SOM mineralization (Bradford et al., 2013; Drake et al., 2013). This mechanism, termed “stoichiometric decomposition,” is feasible as the stoichiometry of glycine is such that its mineralization by microbes would lead to a release of available N into the soil fuelling further microbial growth and SOM decomposition (Hessen et al., 2004). However, the concentration of added glycine was optimized not to stimulate microbial growth (i.e., as confirmed by PLFA analysis, Figure 4C). This mechanism is therefore unlikely to be responsible for the large priming effect observed with the addition of glycine. A second hypothesis is that the supply of available N (in glycine) may have promoted microbial mining of organic P, thereby accelerating SOM mineralization. This theory is supported by evidence of N fertilization in tropical forests stimulating phosphatase activity, possibly as a consequence of extra investment of N in the production of extracellular phosphatases (Olander and Vitousek, 2000; Treseder and Vitousek, 2001). In contrast, however, a study across a semi-arid substrate age gradient in Arizona reported no significant relationship between P availability and priming (Sullivan and Hart, 2013). These conflicting results may be due to difficulties in accurately measuring P availability compared to N. For example, Sullivan and Hart (2013) proposed that sufficient P may have been available across the substrate age gradient to meet microbial demands following C addition and that measurements of labile phosphate may not have reflected total P availability at those sites. There is strong supporting evidence that soil C cycling depends closely on the availability of N and P, and there is evidence that P availability may influence SOC sink strength even in ecosystems traditionally considered N limited (Bradford et al., 2008b, 2013; Hartman and Richardson, 2013). Nevertheless, the mechanisms by which P availability interacts with C and N cycling are poorly resolved and require further investigation.

It should also be considered that our analyses measured the total priming effect resulting from the turnover of non-living SOM and accelerated microbial biomass turnover and did not distinguish between these two sources of priming (Blagodatskaya and Kuzyakov, 2008). It is possible that a proportion of the primed C was derived from the microbial biomass. However, we calculated that after 7 days incubation the amount of primed C respired in response to xylose, vanillin and glycine represented on average 6, 24, and 43% of microbial biomass C, respectively (Supplementary Table 3). Given that estimated microbial turnover times are typically greater than 7 days it is therefore likely that only a small proportion of primed C comes from microbial biomass in the glycine and vanillin amended soils (Chen et al., 2014). However, the response to xylose may be due more to rapid turnover of bacterial biomass triggering “apparent” priming effects (Blagodatskaya and Kuzyakov, 2008).

### Does microbial community composition mediate priming effects?

To test our third hypothesis (H3) we investigated whether the magnitude of priming was determined by differential substrate assimilation among microbial functional groups. The fate of C inputs within microbial communities differed significantly between soils and substrates. Total ^13^C incorporation was highest in soils with greater microbial biomass, and there were also significant differences in ^13^C assimilation by GP and GN bacteria and fungi among soils (Table 5 and Figure 4). Substrate quality was, however, the single strongest factor controlling tracer ^13^C incorporation into GP and GN bacteria (Figure 5 and Table 5). The potential role for GN bacteria in the breakdown and assimilation of labile substrates (vanillin and glycine) and GP bacteria in mineralization of more complex substrates (hemicellulose) identified in this study is consistent with findings from temperate and arctic soil research (Waldrop and Firestone, 2004; Zak and Kling, 2006; Rinnan and Baath, 2009). Fungal assimilation of substrate-derived ^13^C was lower than bacterial assimilation and varied between substrates and soils but there were no clear trends with substrate quality and differences were not related to changes in the amount or proportion of fungi in soils with elevation (Table 5, Figure 5). This could be due to the relatively short incubation time used in priming assays in this study, as fungi (*K*-strategists) typically have slower biomass turnover rates than bacteria (Rousk and Baath, 2011), and are thought to play an important role in priming after added substrates are exhausted by *r*-strategists (Fontaine et al., 2003). Fungal taxa are known to vary in their C resource niches, with some taxa targeting a diverse range of C sources whilst others target specific C resources e.g., lignin degraders (Hanson et al., 2008; Van Der Wal et al., 2013). Differences in fungal assimilation observed here, may therefore mask increases and decreases in assimilation of particular fungal taxa targeting labile and recalcitrant C. These varied capabilities of fungal taxa to utilize C substrates might also explain different responses reported between studies in soils with varied microbial community composition. For example, fungi were important in vanillin mineralization in arctic tundra heath soils but not in wetter tundra soil in Alaska (Zak and Kling, 2006; Rinnan and Baath, 2009).

Overall, these findings demonstrate that significant differences in substrate assimilation between GN and GP bacteria correspond with substrate-related differences in priming effects in all of the tropical forest soils studied here. GP bacteria assimilated a greater proportion of the complex C substrate, hemicellulose (Figure 5C), but a much smaller proportion of the more labile substrates glycine and vanillin (Figures 5B,D). This corresponded with a large positive priming effect for glycine and vanillin but negligible priming in response to hemicellulose (Figures 2F–H). In contrast, GN bacteria metabolized hemicellulose the least of all the substrates but this did not correspond with a significant priming effect. We infer from this that in glycine and vanillin amended soils GP bacteria were mineralizing SOM rather than added substrate, indicating that this functional group may have contributed significantly to enhanced SOM mineralization through priming (Bird et al., 2011; Fontaine et al., 2011). This is consistent with reports that GP bacteria and fungi are more involved in the degradation of organic matter, whilst labile C is predominantly metabolized by GN bacteria (Bird et al., 2011; Koranda et al., 2014). It has been hypothesized that only a part of the microbial community (*K*-strategists) has the physiological capacity to mineralize SOM and that it is the availability of labile C to that component, in competition with *r*-strategists, that determines the magnitude of priming (Fontaine et al., 2011). In this study, the microbial community were functionally defined and quantified by PLFA analysis as GP and GN bacteria and fungi (SP and ECM), and whilst these classifications do not directly correspond to *K*- and *r*-strategists for all phyla, the divisions do broadly overlap: filamentous fungi and GP bacteria are described as *K*-strategists (oligotrophs), slowly and more efficiently targeting recalcitrant C sources including SOM, whilst GN bacteria are classified as *r*-strategists (copiotrophs) targeting simple, labile C substrates (Blagodatskaya et al., 2007; Fierer et al., 2007; De Vries and Shade, 2013; Dungait et al., 2013; Thomson et al., 2013). Our data are consistent with the functional framework of an *r*-*K* spectrum and provide evidence to support hypothesis 3 that K-strategists have the functional capacity to mineralize both fresh C inputs and native SOM and that this explains substrate-related differences in priming effects. However, our data do not support the proposition (H1) that increases in the relative abundance of *K*-strategists will increase SOM mineralization due to priming.

### Microbial carbon-use efficiency and priming

A further metric which it is useful to quantify in relation to C cycling is the CUE of microbial communities (Koranda et al., 2014). Greater microbial CUE in soil from 3025 m asl compared to those from 210 m to 1000 m asl (Figure 6) supports the hypothesis that warm temperatures and low P availability in the lowland soil, have resulted in lower microbial CUE (Whitaker et al., 2014). This lower CUE results in a greater proportion of mineralized C being respired as CO_2_ or invested in extracellular enzymes for P acquisition, rather than being assimilated into microbial biomass (Hartman and Richardson, 2013; Waring et al., 2014). In comparison, in cooler montane soils where microbial N limitation has been described, but P is more available, microbial CUE is higher (Cusack et al., 2011; Fisher et al., 2013) (Figure 6). Microbial CUE has been shown elsewhere to increase with decreasing temperature and increasing nutrient availability (Manzoni et al., 2012; Frey et al., 2013). For example, low microbial growth efficiency was predicted in P-limited lowland tropical soils (Waring et al., 2014) implying that along this gradient, microbial CUE was driven more by P rather than N availability. These results provide further evidence that microbial C metabolism is sensitive to ecosystem N and P availability, with potential feedbacks to soil C turnover in tropical forest ecosystems.

In conclusion, our findings demonstrate that in these tropical forest soils the microbial pathways by which C inputs are processed and the magnitude and direction of priming effects are driven by substrate-specific properties. Substrate quality (i.e., complexity and N content) determined the source of respired CO_2_ (i.e., substrate: SOM) and that partitioning was regulated by the functional capacity of specific microbial groups–in particular *K*-strategists that access labile C inputs and accelerate SOM mineralization. Changes in the complexity and stoichiometry of plant inputs to soil in response to climate change could therefore be important in regulating soil C dynamics and climate-C cycle feedbacks in these C-rich tropical forest soils.

### Conflict of interest statement

The authors declare that the research was conducted in the absence of any commercial or financial relationships that could be construed as a potential conflict of interest.
