# Supplementary material for: Microbial carbon mineralization in tropical lowland and montane forest soils of Peru
Source: Front Microbiol. 2014 Dec 18;5:720. doi: 10.3389/fmicb.2014.00720 (PMC4270188; doi:10.3389/fmicb.2014.00720)
Supplement: Supplementary file 1 [file Table1.DOCX]

***Supplementary Material***

**Supplementary Table 1.**  Supporting data for three-way *anova* of substrate and soil effects on respired substrate-C (*anova* presented in Table 4).

Pair-wise comparisons of substrate and soil effects on substrate-derived C using Tukeys HSD post-hoc tests. Data were subset into 24, 48 and 18 hr data sets. Data for 24 hr only were subset by soil and substrate and analysed by one-way analysis of variance (All one-way ***anova*** were significant at P<0.0001) to investigate significant interactive effects. Data presented in Table 4.

1. **24 hr substrate-derived C, subset by substrate**

| Site | 3400m | 3200m | 3025m | 2720m | 2520m | 2020m | 1850m | 1500m | 1000m | 210m |
| --- | --- | --- | --- | --- | --- | --- | --- | --- | --- | --- |
|  | subset by substrate | | | | | | | | | |
| Xylose | abc | ab | acd | ab | b | abc | abc | d | cd | e |
| Glycine | ab | ab | ab | ab | a | ab | ab | ab | b | c |
| Vanillin | ac | ab | ab | ac | ac | ac | ab | b | ab | c |
| Hemi-cellulose | ab | ab | ab | a | a | ab | a | b | ab | c |
|  | 3400m | 3200m | 3025m | 2720m | 2520m | 2020m | 1850m | 1500m | 1000m | 210m |
|  | subset by soil/site | | | | | | | | | |
| Xylose | b | c | c | c | b | c | c | d | b | b |
| Glycine | a | a | a | a | a | a | a | a | a | a |
| Vanillin | a | ab | ab | a | a | a | ab | b | a | a |
| Hemi-cellulose | b | bc | b | b | b | b | b | c | a | b |

1. **48 and 168 hr substrate-derived C**

| Substrate | 48 hr | 168 hr |
| --- | --- | --- |
| Xylose | b | b |
| Glycine | a | a |
| Vanillin | b | b |
| Hemicellulose | c | a |
| Site | 48 hr | 168 hr |
| 3400m | b | c |
| 3200m | ab | ab |
| 3025m | ab | abc |
| 2720m | ab | ab |
| 2520m | ab | ab |
| 2020m | ab | abc |
| 1850m | ab | ac |
| 1500m | a | b |
| 1000m | ab | abc |
| 210m | c | d |
