# Supplementary material for: Microbial carbon mineralization in tropical lowland and montane forest soils of Peru
Source: Front Microbiol. 2014 Dec 18;5:720. doi: 10.3389/fmicb.2014.00720 (PMC4270188; doi:10.3389/fmicb.2014.00720)
Supplement: Supplementary file 2 [file Table2.DOCX]

***Supplementary Material***

**Supplementary Table 2.** Supporting data for three-way *anova* of substrate and soil effects on respired primed-C (*anova* presented in Table 4).

Pair-wise comparisons of substrate and soil effects on primed C using Tukeys HSD post-hoc tests for data subset by soil and substrate and analysed by one-way analysis of variance (All one-way ANOVAs were significant at P<0.0001). Data presented in Table 4.

1. **Subset by substrate**

|  | 24 hr | | | | 48 hr | | | | 168 hr | | | |
| --- | --- | --- | --- | --- | --- | --- | --- | --- | --- | --- | --- | --- |
| Site | X | G | V | H | X | G | V | H | X | G | V | H |
| 3400 | ab | ab | bc | ab | a | ab | a | ab | a | a | bc | bc |
| 3200 | ab | ab | ab | a | a | ab | a | a | a | a | ab | abc |
| 3025 | cd | a | ab | ab | a | ac | a | ab | a | a | abc | b |
| 2720 | abc | ab | bc | ab | ab | ab | a | ab | a | a | a | abc |
| 2520 | ab | bc | bc | a | a | abc | a | ab | a | a | a | abc |
| 2020 | d | ab | bc | ab | a | bd | a | ab | a | a | a | abc |
| 1850 | abcd | ab | bc | bc | ab | abc | a | a | a | a | abc | ac |
| 1500 | acd | ab | ab | d | a | c | a | a | a | a | abc | a |
| 1000 | abcd | ab | ab | cd | ab | abc | a | ab | a | a | abc | ac |
| 210 | b | c | c | a | b | d | b | b | a | b | c | abc |

1. **Subset by soil/site**

|  |  | 3400 | 3200 | 3025 | 2720 | 2520 | 2020 | 1850 | 1500 | 1000 | 210 |
| --- | --- | --- | --- | --- | --- | --- | --- | --- | --- | --- | --- |
| 24 hr | X | b | c | c | d | c | b | b | c | c | b |
|  | G | a | a | a | a | a | a | a | a | a | a |
|  | V | b | a | a | b | a | b | ab | a | a | ab |
|  | H | c | b | b | c | b | c | c | b | b | c |
| 48 hr | X | c | c | c | c | c | c | c | d | d | c |
|  | G | a | a | a | a | a | a | a | a | a | a |
|  | V | a | a | a | a | a | a | a | b | b | a |
|  | H | b | b | b | b | b | b | b | c | c | b |
| 168 hr | X | b | c | c | d | c | b | c | d | d | d |
|  | G | a | a | a | a | a | a | a | a | a | a |
|  | V | b | b | b | b | b | a | a | b | b | b |
|  | H | b | c | c | c | c | b | b | c | c | c |
