# Supplementary material for: Microbial carbon mineralization in tropical lowland and montane forest soils of Peru
Source: Front Microbiol. 2014 Dec 18;5:720. doi: 10.3389/fmicb.2014.00720 (PMC4270188; doi:10.3389/fmicb.2014.00720)
Supplement: Supplementary file 3 [file Table3.DOCX]

***Supplementary Material***

**Supplementary Table 3.** Calculation of primed C relative to microbial biomass C

In order to assess the magnitude of primed C in relation to microbial biomass C, we used data from Whitaker et al (2014) for 8 of the field sites where 0-10 cm depth soil samples corresponded with only the organic horizon of the soil.

| Elevation (m asl) | Microbial biomass C (µg C g^-1^ soil dwt) | Xylose primed C (µg C g^-1^ soil dwt) | Glycine primed C (µg C g^-1^ soil dwt) | Vanillin primed C (µg C g^-1^ soil dwt) | Xylose  primed C as % of micC | Glycine  primed C as % of micC | Vanillin primedC as % of micC |
| --- | --- | --- | --- | --- | --- | --- | --- |
| 1500 | 1244.84 | 124.97 | 722.77 | 353.58 | 10.04 | 58.06 | 28.4 |
| 1850 | 1911.77 | 63.78 | 519.84 | 310.97 | 3.34 | 27.19 | 16.27 |
| 2020 | 1663.29 | 97.34 | 635.81 | 411.53 | 5.85 | 38.23 | 24.74 |
| 2520 | 1130.75 | 99.59 | 663.01 | 421.44 | 8.81 | 58.63 | 37.27 |
| 2720 | 1181.84 | 78.18 | 665.46 | 417.6 | 6.62 | 56.31 | 35.34 |
| 3025 | 2261.59 | 114.93 | 723.44 | 358.97 | 5.08 | 31.99 | 15.87 |
| 3200 | 1371.85 | 58.5 | 628.41 | 372.43 | 4.26 | 45.81 | 27.15 |
| 3400 | 1817.38 | 56.84 | 522.61 | 166.22 | 3.13 | 28.76 | 9.15 |
| **Average** |  |  |  |  | **5.9 %** | **43 %** | **24 %** |
